# Factors Associated with Mental Health among International Students during the COVID-19 Pandemic in South Korea

**DOI:** 10.3390/ijerph182111381

**Published:** 2021-10-29

**Authors:** Hae Ran Kim, Eun Jung Kim

**Affiliations:** 1 Department of Nursing, College of Medicine, Chosun University, Gwangju 61452, Korea; rahn00@chosun.ac.kr; 2 Department of Nursing, Honam University, 417 Eodeung-daero, Gwangsan-gu, Gwangju 62399, Korea

**Keywords:** COVID-19, international students, sleep problem, anxiety, depression

## Abstract

The purpose of this study was to investigate mental health problems among international students in South Korean universities during the COVID-19 pandemic, as well as to identify the factors that affect their mental health. A total of 488 international students living in South Korea participated in a web-based survey. The questionnaire was created using the Google Forms platform, and a link to the questionnaire was shared through social media. Multiple logistic regression analysis was conducted to analyze the data. The prevalence rates of sleep problems, anxiety, and depression among international students were 47.1%, 39.6%, and 49%, respectively. The prevalence of mental health problems was higher among participants who were male, living with someone, residents of a rural area, and earning a higher income. The following variables were found to contribute to the prevalence of mental health problems: undergraduate student status, good understanding of the Korean language, longer hours of media usage, and experiences related to COVID-19 infection. A collaborative effort between the government and universities to manage the mental health of international students could promote the mental health of these students.

## 1. Introduction

Since the World Health Organization (WHO) declared an international public health emergency in March 2020, coronavirus disease 2019 (COVID-19) has changed the lives of people worldwide [1]. Due to the prolonged COVID-19 pandemic, many countries have implemented control measures to prevent the spread of infection, such as imposing travel restrictions on foreign nationals and closing down public places and public transportation systems [2,3].

South Korea was the third country to report a confirmed case of COVID-19 outside of China [4]. Since February 2020, the Korea Disease Control and Prevention Agency (KDCA) has implemented stringent social distancing measures and restricted everyday socioeconomic activities [5]. In accordance with government guidelines, information regarding COVID-19 was distributed in real time through news and social media platforms. After the first case of COVID-19 was reported in South Korea, the Korean Ministry of Education postponed the beginning of the school year four times, and a phased online approach was put into place for the start of the term [6]. Similarly, colleges canceled on-campus classes that were scheduled to start on 2 March 2020; as universities were unable to conduct in-person classes, they implemented emergency remote learning services and offered classes online [7]. The unexpected circumstances regarding the COVID-19 control measures may have a detrimental effect on the mental health of university students. Studies have shown that the most common mental health problems among students of higher education are sleep problems, anxiety, and depression [8]. After the onset of the COVID-19 pandemic, nearly half of U.S. university students reported sleep problems and anxiety [9], while 56.8% of Chinese university students reported depression [10]. A study conducted in the United States early in the pandemic found that financial problems, large amounts of misinformation, and the social stigma of people diagnosed with COVID-19 were associated with feelings of fear and anxiety among university students [11]. A study of university students in China reported that financial problems and the threat of infection during the COVID-19 pandemic impacted the students’ anxiety and depression levels [12].

Compared to domestic students, international students face greater difficulties in maintaining their mental health during the COVID-19 pandemic. The following factors are likely to exacerbate the adverse effect on their mental health: differences in the ability to absorb information due to language barriers, health status, economic pressure, living abroad status, adaptation to the host country’s social culture, and the lack of access to family social support systems [13,14,15]. Host countries have neglected the social support of international students, who constitute a small percentage of university student populations. For example, certain universities in South Korea closed down without regard for the difficulties that international students would face in returning to their home countries due to border closures, reductions of international flights, and the risk of exposure to infection on flights [16]. The mental health of international students who remain in South Korea without being able to return to their home countries must be examined. A study conducted in France reported that international students are more likely than French university students to have one or more of five mental health problems, including depression symptoms and anxiety [17]. A study of international students in the United Kingdom and the United States found that the stayers were more likely to experience sleep problems, anxiety, and depression than the returnees [13]. These studies can measure the impact of unexpected emergencies on the mental health of international students, as well as help host countries establish effective intervention strategies for the promotion of their mental health. However, no study to date in South Korea has investigated the mental health status of international students remaining in the country during the COVID-19 pandemic. Therefore, the aims of this study were twofold: (1) to use an online survey to examine the prevalence of sleep problems, anxiety, and depression among international students living in South Korea during the COVID-19 pandemic, and (2) to identify the factors that affect these mental health problems.

## 2. Materials and Methods

### 2.1. Data Source

The survey was conducted from 10 December to 31 December 2020. The target population comprised international students of universities located in various regions of South Korea. We recruited participants through the online bulletin board of an affiliated university. Participants then shared the survey with their family, friends, and colleagues residing in South Korea. Google Forms was used to collect information on the students’ general characteristics, sleep problems, anxiety, and depression.

### 2.2. Sampling Technique

The snowball sampling technique was used to collect information. An informed consent form was included in the online questionnaire, and each participant read the consent form and signed it to indicate their agreement to participate in the survey. Participants were asked to share the online questionnaire with their acquaintances through personal and institutional social media channels.

### 2.3. Measures

The questionnaire included items regarding gender (male or female), age (20–24, 25–30, or >30), currently studying (undergraduate or postgraduate degree), living arrangement (living alone or living with someone), monthly income (none, ≤USD 883, or >USD 883), location of university (urban or rural), understanding level of Korean media (good or poor), daily average number of hours of media usage about COVID-19 (<2 h or ≥2 h), COVID-19 symptom experience (no or yes), and experience of clinical care due to contact with a confirmed case of COVID-19 (no or yes). Regarding living arrangements, “living alone” was classified as “yes” for those living alone and “no” for those living with at least one other person, including family members. The location of a university was defined as urban if it was located in a region of South Korea that was designated as a metropolitan city with a population of at least 500,000 people, and the location of all other universities was defined as a rural area. To evaluate their understanding of the Korean media, participants were asked: “How well do you understand information in the Korean media?” Participants could respond with one of the following choices: very incomprehensible, incomprehensible, neutral, comprehensible, and very comprehensible. The responses “comprehensible” and “very comprehensible” were classified as “good” and all other responses were classified as “poor”. With regard to the participants’ COVID-19 symptom experience, having experienced one or more of the following symptoms was classified as “yes”, and having no experience was classified as “no”: fever of 38 °C or higher for at least one day, chills, headache, muscle aches, cough, difficulty breathing, vertigo, runny nose, and sore throat. Regarding the participants’ experience of clinical care due to contact with a confirmed case of COVID-19, having experience of one or more of the following was classified as “yes” and having no experience was classified as “no”: diagnostic tests, isolation, and hospitalization during the pandemic due to contact with a confirmed or suspected case of COVID-19.

In this study, mental health was measured as sleep problems, anxiety, and depression. First, sleep problems were measured with questions such as the following: “During the COVID-19 pandemic, have you had symptoms such as difficulty falling asleep, frequent waking at night, or too much sleep in the past two weeks compared to before the pandemic?” The participants could answer “yes” or “no”. Second, levels of anxiety were measured using the Generalized Anxiety Disorder Scale (GAD-7). The GAD-7 is a measurement tool that is widely used for the efficient measurement of anxiety symptoms [18]. The GAD-7 includes items relating to seven core symptoms and assesses the frequency with which respondents experienced the symptoms in the last two weeks. The responses range from “not at all” (0) to “nearly every day” (3), and the total score for the seven items ranges from 0 to 21. In this study, the levels of anxiety were categorized as “without anxiety symptoms” (0–4), “mild” (5–9), “moderate” (10–14), and “severe” (15–21) [19]. Third, depression levels were measured using the Patient Health Questionnaire (PHQ-9). The PHQ-9 is a useful and easy-to-use tool for the screening and prediction of depression [20]. The PHQ-9 consists of items that assess the frequency with which nine depression symptoms were experienced in the last two weeks. The participants can choose a response that ranges from “not at all” (0) to “nearly every day” (3), and the total score for the nine items ranges from 0 to 27. The levels of depression for the study were categorized as “without depression symptoms” (0–4), “mild” (5–9), “moderate” (10–14), “moderately severe” (15–19), and “severe” (20–27) [21].

### 2.4. Data Analysis

The data collected using the Google Forms software were analyzed using the IBM SPSS Statistics for Windows, version 24 (IBM Corp., Armonk, NY, USA). Descriptive statistics were used to explain the general characteristics of the respondents and the prevalence of mental health problems. A chi-square test was used to analyze the differences between sleep problems, anxiety, and depression according to the respondents’ general characteristics. Multiple logistic regression was used to identify the general characteristic variables that influence the state of the respondents’ mental health. This study classified mental health variables into two categories and identified the variables that affect the respondents’ sleep problems, anxiety, and depression. Sleep problems were classified as “yes” and “no”, while depression and anxiety were classified as “no” for the level “without symptoms” and classified as “yes” for the levels “mild, moderate, moderately severe, and severe”. Odds ratios (ORs) with 95% confidence intervals (CIs) were reported. The Hosmer–Lemeshow test was used to assess the model fit of the multiple logistic regressions.

### 2.5. Ethical Issues

The study was conducted in accordance with the Declaration of Helsinki. This study was formally approved by the Ethical Clearance Committee of Honam University, Korea (1041223-202008-HR-10). The participants responded to the questionnaire anonymously after completing an informed consent form in the first section of the online survey. The consent form provided all participants with information regarding the purpose of the study, confidentiality of the data, and the right to withdraw their participation without prior justification.

## 3. Results

### 3.1. Frequency for General Characteristic Variables

Table 1 presents information on the general characteristic variables of international students in South Korea. Regarding the respondents’ gender and age, 41.8% were male and 58.2% were female, and most respondents (63.9%) were in the 25–30 years age group. Of the respondents, 32.6% were undergraduate students, and 67.4% were graduate students. Regarding the respondents’ living arrangements and monthly income, 27.3% were living with someone and 72.3% earned a monthly income of ≤USD 883. Regarding the respondents’ location of residence, 23.2% resided in an urban area and 76.8% resided in a rural area. Of the respondents, 56.6% had a good level of understanding of Korean media while 43.4% had a poor level of understanding, and 29.5% answered that they used the media to obtain COVID-19-related information for at least two hours per day. Of the respondents, 37.1% reported having experienced respiratory symptoms, while 15.0% reported having experienced clinical care due to contact with a confirmed case of COVID-19 (Table 1).

### 3.2. Prevalence of Sleep Problems, Anxiety, and Depression among International Students during the COVID-19 Pandemic

Table 2 lists the prevalence of sleep problems, anxiety, and depression among international students. Sleep problems were reported by 47.1% (*n* = 230) of the international student participants. According to the results of the GAD-7, anxiety symptoms were reported by 39.6% of participants, with 33.2% and 6.4% having mild and moderate levels of anxiety, respectively. In addition, according to the results of the PHQ-9, depression symptoms were reported by 49% of participants, with 34%, 8.0%, and 7.0% having mild, moderate, and moderate to severe levels of depression, respectively (Table 2).

### 3.3. Bivariate Analyses Comparing Sociodemographic Characteristics and Mental Health

Sleep problems were most prevalent among students with the following characteristics: male (χ^2^ = 14.70, *p* < 0.001), aged 20–24 (χ2 = 7.30, *p* = 0.026), undergraduate (χ^2^ = 7.40, *p* = 0.007), living with someone (χ^2^ = 88.98, *p* < 0.001), monthly income of >USD 883 (χ^2^ = 26.99, *p* < 0.001), rural residence (χ^2^ = 9.40, *p* = 0.002), good understanding level of Korean media (χ^2^ = 7.45, *p* = 0.006), COVID-19 symptom experience (χ^2^ = 64.24, *p* < 0.001), and experience of clinical care due to contact with a confirmed case of COVID-19 (χ^2^ = 60.51, *p* < 0.001). Anxiety was most prevalent among students with the following characteristics: male (χ^2^ = 4.51, *p* = 0.034), undergraduate (χ^2^ = 12.81, *p* < 0.001), living with someone (χ^2^ = 14.64, *p* < 0.001), urban residence (χ^2^ = 9.86, *p* = 0.002), good understanding level of Korean media (χ^2^ = 23.30, *p* < 0.001), at least two hours of COVID-19-related media usage per day (χ^2^ = 4.16, *p* = 0.041), experience of COVID-19-related symptoms (χ^2^ = 13.85, *p* < 0.001), and experience of clinical care due to contact with a confirmed case of COVID-19 (χ^2^ = 46.00, *p* < 0.001). Depression was most prevalent among students with the following characteristics: male (χ^2^ = 41.50, *p* < 0.001), undergraduate (χ^2^ = 13.66, *p* < 0.001), living with someone (χ^2^ = 53.20, *p* < 0.001), monthly income of >USD 883 (χ^2^ = 68.96, *p* < 0.001), rural residence (χ^2^ = 8.20, *p* = 0.004), good understanding level of Korean media (χ^2^ = 15.90, *p* < 0.001), experience of COVID-19-related symptoms (χ^2^ = 28.26, *p* < 0.001), and experience of clinical care due to contact with a confirmed case of COVID-19 (χ^2^ = 17.02, *p* < 0.001) (Table 3).

### 3.4. Factors Associated with Mental Health Problems among International Students during the COVID-19

After adjustment for confounding factors, sleep problems were associated with young age (aOR = 0.15, 95% CI = 0.05–0.45, *p* = 0.001), living with someone (aOR = 13.68, 95% CI = 7.05–26.55, *p* < 0.001), rural residence (aOR = 2.36, 95% CI = 1.08–5.15, *p* = 0.031), COVID-19 symptoms experience (aOR = 8.72, 95% CI = 4.72–16.11, *p* < 0.001), and clinical experience due to contact with a confirmed case of COVID-19 (aOR = 27.78, 95% CI = 10.17–75.86, *p* < 0.001) (Table 4). Anxiety was associated with a lower education level (aOR = 0.36, 95% CI = 0.22–0.58, *p* < 0.001), living with someone (aOR = 1.72, 95% CI = 1.06–2.79, *p* = 0.027), urban residence (aOR = 0.34, 95% CI = 0.20–0.60, *p* = 0.027), good understanding level of Korean media (aOR = 0.31, 95% CI = 0.19–0.52, *p* < 0.001), and clinical experience due to contact with a confirmed case of COVID-19 (aOR = 8.29, 95% CI = 4.31–15.94, *p* < 0.001) (Table 5). Depression was associated with male sex (aOR = 0.32, 95% CI = 0.20–0.50, *p* = 0.001), lower education level (aOR = 0.52, 95% CI = 0.31–0.87, *p* = 0.013), living with someone (aOR = 2.93, 95% CI = 1.71–5.01, *p* < 0.001), higher income (aOR = 4.23, 95% CI = 1.86–9.60, *p* = 0.001, for ≤USD 883, and aOR = 29.39, 95% CI = 9.71–88.95, *p* < 0.001, for >USD 883), longer times of media usage about COVID-19 (aOR = 2.28, 95% CI = 1.34–3.88, *p* = 0.002), COVID-19 symptoms experience (aOR = 2.44, 95% CI = 1.44–4.13, *p* = 0.001), and clinical experience due to contact with a confirmed case of COVID-19 (aOR = 4.60, 95% CI = 2.24–9.44, *p* < 0.001) (Table 6).

## 4. Discussion

This study was conducted to investigate the prevalence of sleep problems, anxiety, and depression among international students living in South Korea during the first year of the COVID-19 pandemic as well as to examine the risk factors for these mental health problems. According to the results of this study, sleep problems were reported by 47.1% of respondents, while 33.2% reported mild anxiety and 6.4% reported moderate anxiety. Mild depression and moderate to severe depression were reported by 34% and 15% of respondents, respectively. These figures are similar to previous study of university students during the COVID-19 pandemic, which found that 42% of U.S. university students experienced sleep problems [9]. These results differ from what was observed in previous studies measuring levels of anxiety and depression using the GAD-7 and PHQ-9. According to a previous study of U.S. university students [11], approximately 33% and 38% of students had mild and moderate to severe levels of anxiety, respectively, while about 32% and 48% of students had mild and moderate to severe levels of depression, respectively. In other studies, depression was reported by approximately 50% of general university students in South Korea [22], and a moderate to severe level of depression was reported by about 39% of Chinese international students remaining in South Korea [23]. However, severe levels of anxiety among international students were not observed in the results of this study, and the rate of students with a moderate or greater level of depression was lower than that of previous studies. These differences could be related to the methodological differences in recruitment strategies, which could lead to the sociodemographic characteristics of study subjects. Moreover, the differences could be related to the political measures for infectious disease management depending on when the data were collected. The snowball sampling technique, which was used because of the social distancing measures that were implemented during the pandemic, may have yielded different results from those of previous studies because it is not based on the random selection of samples [24]. In this study, data were collected in December 2020 and, in the studies cited above, data were collected between April and June. The first half of 2020 was a period in which the fatality rate of COVID-19 had greatly increased [25], and the second half of 2020 was a period in South Korea in which the spread of infection was being mitigated with solid infection control strategies [26]. This could explain the lower prevalence rates of anxiety and depression among international students compared to those of previous studies. However, as in previous studies, the prevalence rates of mental health problems were higher among international students than among the general population [27]. The prevalence rates of anxiety and depression in this study were higher than those of a South Korean national survey conducted around the same time, as the latter reported that the prevalence of anxiety was 18.9% and the prevalence of depression was 22.1% [28]. Moreover, the prevalence rates of anxiety and depression reported in this study were higher than those reported by South Korea’s National Center for Disaster Trauma (NCT), which found that the prevalence rates of moderate to severe anxiety and depression among general patients were 8.0% and 14.3%, respectively, between March and November 2020 [29]. This study’s findings suggest that international students in South Korea are experiencing higher prevalence rates of mental health problems during the current global pandemic compared to the general population of South Korea. Therefore, South Korean health policies and host universities should provide international students with mental health management and palliative care programs.

The prevalence of sleep problems was higher in the 20–24 years age group. The association between sleep problems and age is consistent with the results of a study on university students that was conducted during the pandemic [30]. Considering that the study participants are international students, younger students tend to be more concerned about education costs and an educational future made uncertain by a pandemic when compared to older students [31]. In addition, the younger the students are, the more they engage in social media [32], which may lead to sleep problems. In this context, the above could explain the greater prevalence rates of anxiety and depression among younger undergraduate students than among graduate students. Campus mental health services that provide virtual counseling via social media could help international undergraduate students, including freshman students, manage their mental health [33].

This study reported several findings that differ from those of previous studies conducted during the pandemic. First, contrary to a study that reported that family is a mitigating factor against mental health problems [34], this study found that living with someone was associated with a higher prevalence of sleep problems, anxiety, and depression among international students. As international students have a weaker social support base [27], they may have greater fears and concerns than the general population regarding the health of family members or cohabitants, which may in turn be linked to mental health problems [23]. Though beyond the purview of this study, it is highly likely that international graduate students living in South Korea with young children would report anxiety and depression due to the risks and concerns of infection during the pandemic [35]. Ensuring that international students are aware of the social support that exists and is available for their family or cohabitants during the pandemic could provide a basis to address the detrimental state of their mental health. Second, the prevalence of sleep problems, anxiety, and depression was higher in men than in women. As with a study conducted prior to the COVID-19 pandemic [36], this is contrary to previous studies that investigated general university students [37]. In a systematic review and meta-analysis on COVID-19, there was no difference in the prevalence of anxiety and depression across genders [38]. Students’ educational future and life satisfaction were made uncertain by university closures, the disruption of economic activities, and the severance of social relationships following the lockdown, and this may have had a greater impact on male international students than on female international students [31]. Long-term disruptions in employment were reported to be an aggravating factor for COVID-19-related mental health problems in men [39]. Although worsening mental health problems are more likely to lead to suicide in men [40], the mental health of men is overlooked more than that of women. Therefore, particular attention should be paid to male international students in campus mental health care programs. Third, sleep problems were higher in rural students and anxiety was higher in urban students. The rapid spread of COVID-19 across the regions of South Korea in the second half of 2020 [6] and the inadequate policies of small and medium-sized rural universities regarding the continuation of students’ studies may have had a detrimental effect on the mental health of international students [41]. Further, although urban cities had a solid response to the COVID-19 pandemic, they also had a high population density; as a result, a high perceived risk of infection may have been associated with higher levels of anxiety among urban university students [42]. Finally, in contrast to a previous study [12], this study found that higher income was associated with depression among international students. At the time of the survey, when the government relaxed its infection control measures, international students may have chosen to engage in economic activities to cope with the high levels of psychological burden they experienced. For example, international students suffering from depression due to experiencing economic pressure are more likely to be employed than those who are not [14]. Further studies need to be conducted on the association between income and mental health problems caused by COVID-19.

A good understanding of the Korean language and longer hours of media usage can be understood in the same context. Regardless of whether the information about COVID-19 obtained through the media is reliable, excessive information and higher frequency and duration of media use may affect psychological strain [16]. Psychological strain may have exacerbated the poor mental health of international students. The effective delivery of accurate information through the media is necessary in managing the mental health of international students. Similar to previous study of international students and university students [23], this study found that having experienced COVID-19-related respiratory symptoms and having experience of clinical care in the form of diagnostic tests, isolation, and hospitalization due to contact with a confirmed or suspected case of COVID-19 were associated with mental health problems among international students. According to a systematic review and meta-analysis study, individuals who had respiratory symptoms during the COVID-19 pandemic were more than three times more likely to experience depression and more than four times more likely to experience anxiety than those who did not [38]. Experiences of isolation, hospitalization, and stigma due to COVID-19 infection may lead to mental health problems [43]. Local communities and universities must continue to strive to remove the stigma of illness for international students. Furthermore, campus health managers should provide resources for the psychological support and interventions of international students who need to be isolated and hospitalized after experiencing respiratory symptoms or coming into contact with a case of COVID-19.

There are limitations to consider when interpreting the results of this study. First, self-report questionnaires may be associated with recall bias. However, self-reports based on validated screening tools are commonly used as a cost-effective approach. In addition, the reported prevalence of sleep, anxiety, and depression may differ from the actual prevalence of these problems because respondents may have chosen socially desirable responses. Second, the snowball sampling technique was used in this study. Therefore, it may not be possible to draw statistical inferences from a sample that can be applied to the population because the respondents were not randomly selected. However, the snowball sampling technique was a useful method for investigating the prevalence of mental health problems among international students while maintaining social distancing during the COVID-19 pandemic [25]. Longitudinal studies are needed to examine the trajectory of mental health status in international university students in the post-pandemic era. Third, the respondents’ computer skills may have influenced the responses to the online self-report questionnaire. However, conducting a direct investigation was considered to be inappropriate due to the risk of COVID-19 infection. Fourth, data regarding the clinical diagnosis of mental health problems were not collected. Therefore, this study’s findings should only be viewed as an evaluation of the trajectory and degree of sleep problems, anxiety, and depression. However, using validated standardization tools such as the GAD-7 and PHQ-9 was an appropriate method for exploring the situation in a cost-effective manner [18,20]. Lastly, this study could not assess the severity of sleep problems, anxiety symptoms, and depression symptoms that were solely caused by the pandemic crisis because of the lack of pre-COVID-19 data.

## 5. Conclusions

This study is the first to provide empirical evidence suggesting that a significant number of international students in South Korea are suffering from sleep problems, anxiety, and depression as the pandemic persists. Mental health problem takes a high toll on individuals and societies. Given the prevalence of mental health problems in international students, mental health services for this population amid the pandemic should include periodic evaluation of mental health to ensure early identification of students with severe stress, anxiety, and depression and psychiatric assessment and treatment when necessary. In contrast to previous studies, this study found that international students who are male, living with someone, residents of a rural area, and earning a higher income had a higher prevalence of mental health problems. These findings were newly discovered in this study. In addition, several risk factors for mental health problems were identified, such as having experienced COVID-19-related respiratory symptoms and experience of clinical care due to contact with a confirmed or suspected case of COVID-19. The current research findings could help identify the international students who are more vulnerable to experiencing mental health problems during the COVID-19 pandemic, as well as help provide information for the public health strategies that should be established by South Korean policies and universities in the future to improve the mental health of international students. To minimize the prevalence of mental health problems among international students, the South Korean government and universities must collaborate to provide international students with prompt and effective psychological support.

## Figures and Tables

**Table 1:** Frequencies of general characteristic variables (*N* = 488).

Characteristics	Frequency	Percentage
Sex		
Male	204	41.8
Female	284	58.2
Age (years)		
20–24	110	22.5
25–30	312	63.9
>30	66	13.6
Currently studying		
For an undergraduate	159	32.6
For a postgraduate	329	67.4
Living arrangement		
Living alone	355	72.7
Living with someone	133	27.3
Monthly income		
None	53	10.9
≤USD 883	353	72.3
>USD 883	82	16.8
Location of university		
Urban	113	23.2
Rural	375	76.8
Understanding level of Korean media		
Good	276	56.6
Poor	212	43.4
Daily average number of hours of media usage about COVID-19		
<2 h	344	70.5
≥2 h	144	29.5
COVID-19 symptom experience		
No	307	62.9
Yes	181	37.1
Experience of clinical care due to contact with a confirmed case of COVID-19		
No	415	85.0
Yes	73	15.0

COVID-19 symptom experience indicates having experienced one or more of the following symptoms: fever of 38 °C or higher for at least one day, chills, headache, muscle aches, cough, difficulty in breathing, vertigo, runny nose, and sore throat. Experience of clinical care due to contact with a confirmed case of COVID-19 indicates having one or more experiences of diagnostic tests, isolation, and hospitalization during the COVID-19 pandemic due to coming into contact with a confirmed or suspected case of COVID-19.

**Table 2:** Prevalence of sleep problems, anxiety, and depression among international students during the COVID-19 (*N* = 488).

Variables	Classification	Frequency	Percentage
Sleep problems	No	258	52.9
	Yes	230	47.1
Anxiety (GAD-7)	Without anxiety symptoms (0–4)	295	60.5
	With mild anxiety symptoms (5–9)	162	33.2
	With moderate anxiety symptoms (10–13)	31	6.4
Depression (PHQ-9)	Without depression symptoms (0–4)	249	51.0
	With mild depression symptoms (5–9)	166	34.0
	With moderate depression symptoms (10–14)	39	8.0
	With moderate to severe depression symptoms (15–19)	34	7.0

GAD-7: Generalized Anxiety Disorder Scale-7; PHQ-9: Patient Health Questionnaire.

**Table 3:** Bivariate analyses comparing sociodemographic characteristics and mental health (*N* = 488).

Variables	Sleep Problems				Anxiety (GAD-7)				Depression (PHQ-9)			
	No	Yes	χ^2^	*p*	No	Yes	χ^2^	*p*	No	Yes	χ^2^	*p*
Gender			14.70	<0.001			4.51	0.034			41.50	<0.001
Male	87 (42.6)	117 (57.4)			112 (54.9)	92 (45.1)			69 (33.8)	135 (66.2)		
Female	171 (60.2)	113 (39.8)			183 (64.4)	101 (35.6)			180 (63.4)	104 (36.6)		
Age (years)			7.30	0.026			3.76	0.153			0.82	0.663
20–24	54 (49.1)	56 (50.9)			66 (60.0)	44 (40.0)			53 (48.2)	57 (51.8)		
25–30	159 (51.0)	153 (49.0)			196 (62.8)	116 (37.2)			164 (52.6)	148 (47.4)		
>30	45 (68.2)	21 (31.8)			33 (50.0)	33 (50.0)			32 (48.5)	34 (51.5)		
Currently studying			7.40	0.007			12.81	<0.001			13.66	<0.001
For an undergraduate	70 (44.0)	89 (56.0)			78 (49.1)	81 (50.9)			62 (39.0)	97 (61.0)		
For a postgraduate	188 (57.1)	141 (42.9)			217 (66.0)	112 (34.0)			187 (56.8)	142 (43.2)		
Living arrangement			88.98	<0.001			14.64	<0.001			53.20	<0.001
Living alone	234 (65.9)	121 (34.1)			233 (65.6)	122 (34.4)			217 (61.1)	138 (38.9)		
Living with someone	24 (18.0)	109 (82.0)			62 (46.6)	71 (53.4)			32 (24.1)	101 (75.9)		
Monthly income			26.99	<0.001			4.82	0.090			68.96	<0.001
None	23 (43.4)	30 (56.6)			28 (52.8)	25 (47.2)			36 (67.9)	17 (32.1)		
≤USD 883	211 (59.8)	142 (40.2)			224 (63.5)	129 (36.5)			205 (58.1)	148 (41.9)		
>USD 883	24 (29.3)	58 (70.7)			43 (52.4)	39 (47.6)			8 (9.8)	74 (90.2)		
Location of university			9.40	0.002			9.86	0.002			8.20	0.004
Urban	74 (65.5)	39 (34.5)			54 (47.8)	59 (52.2)			71 (62.8)	42 (37.2)		
Rural	184 (49.1)	191 (50.9)			241 (64.3)	134 (35.7)			178 (47.5)	197 (52.5)		
Understanding level of Korean media			7.45	0.006			23.30	<0.001			15.90	<0.001
Good	131 (47.5)	145 (52.5)			141 (51.1)	135 (48.9)			119 (43.1)	157 (56.9)		
Poor	127 (59.9)	85 (40.1)			154 (72.6)	58 (27.4)			130 (61.3)	82 (38.7)		
Daily average number of hours of media usage about COVID-19			0.68	0.411			4.16	0.041			3.54	0.060
<2 h	186 (54.1)	158 (45.9)			218 (63.4)	126 (36.6)			185 (53.8)	159 (46.2)		
≥2 h	72 (50.0)	72 (50.0)			77 (53.5)	67 (46.5)			64 (44.4)	80 (55.6)		
COVID-19 symptom experience			64.24	<0.001			13.85	<0.001			28.26	<0.001
No	205 (66.8)	102 (33.2)			205 (66.8)	102 (33.2)			185 (60.3)	122 (39.7)		
Yes	53 (29.3)	128 (70.7)			90 (49.7)	91 (50.3)			64 (35.4)	117 (64.6)		
Experience of clinical care due to contact with a confirmed case of COVID-19			60.51	<0.001			46.00	<0.001			17.02	<0.001
No	250 (60.2)	165 (39.8)			277 (66.7)	138 (33.3)			228 (54.9)	187 (45.1)		
Yes	8 (11.0)	65 (89.0)			18 (24.7)	55 (75.3)			21 (28.8)	52 (71.2)		

Values are expressed as frequency (%). aOR: adjusted odds ratio; CI: confidence interval.

**Table 4:** Factors associated with sleep problems among international students during the COVID-19 (*N* = 488).

Variables	Sleep Problems			
	B	*p*	aOR	95% CI
Age (years)				
20–24	1.00		1.00	
25–30	0.82	0.801	1.09	0.58–2.05
>30	−1.93	0.001	0.15	0.05–0.45
Living arrangement				
Living alone	1.00		1.00	
Living with someone	2.62	<0.001	13.68	7.05–26.55
Location of university				
Urban	1.00		1.00	
Rural	0.86	0.031	2.36	1.08–5.15
COVID-19 symptoms experience				
No	1.00		1.00	
Yes	2.17	<0.001	8.72	4.72–16.11
Experience of clinical care due to contact with a confirmed case of COVID-19				
No	1.00		1.00	
Yes	3.32	<0.001	27.78	10.17–75.86

Values are expressed as frequency (%). aOR: adjusted odds ratio; CI: confidence interval.

**Table 5:** Factors associated with depression among international students during the COVID-19 (*N* = 488).

Variables	Anxiety (GAD-7)			
	B	*p*	aOR	95% CI
Currently studying				
For an undergraduate	1.00		1.00	
For a postgraduate	−1.03	<0.001	0.36	0.22–0.58
Living arrangement				
Living alone	1.00		1.00	
Living with someone	0.54	0.027	1.72	1.06–2.79
Location of university				
Urban	1.00		1.00	
Rural	−1.08	<0.001	0.34	0.20–0.60
Understanding level of Korean media				
Good	1.00		1.00	
Poor	−1.16	<0.001	0.31	0.19–0.52
Experience of clinical care due to contact with a confirmed case of COVID-19				
No	1.00		1.00	
Yes	2.11	<0.001	8.29	4.31–15.94

Values are expressed as frequency (%). aOR: adjusted odds ratio; CI: confidence interval.

**Table 6:** Factors associated with depression among international students during the COVID-19 (*N* = 488).

Variables	Depression (PHQ-9)			
	B	*p*	aOR	95% CI
Gender				
Male	1.00		1.00	
Female	−1.15	<0.001	0.32	0.20–0.50
Currently studying				
For an undergraduate	1.00		1.00	
For a postgraduate	−0.65	0.013	0.52	0.31–0.87
Living arrangement				
Living alone	1.00		1.00	
Living with someone	1.08	<0.001	2.93	1.71–5.01
Monthly income				
None	1.00		1.00	
≤USD 883	1.44	0.001	4.23	1.86–9.60
>USD 883	3.38	<0.001	29.39	9.71–88.95
Daily average number of hours of media usage about COVID-19				
<2 h	1.00		1.00	
≥2 h	0.82	0.002	2.28	1.34–3.88
COVID-19 symptoms experience				
No	1.00		1.00	
Yes	0.89	0.001	2.44	1.44–4.13
Experience of clinical care due to contact with a confirmed case of COVID-19				
No	1.00		1.00	
Yes	1.53	<0.001	4.60	2.24–9.44

Values are expressed as frequency (%). aOR: adjusted odds ratio; CI: confidence interval.

## Data Availability

The data presented in this study are available on request from the corresponding author.
